# Fetal Bradycardia Prompting the Diagnosis and Management of Parental Long QT Syndrome

**DOI:** 10.1161/CIRCEP.124.013360

**Published:** 2025-12-02

**Authors:** Kiruthika Ananthan, Sian Chivers, Will Regan, Antonio de Marvao, Trisha Vigneswaran, Eric Rosenthal, Vita Zidere, Tessa Homfray, Catherine Williamson, John M. Simpson, Rachel Bastiaenen, John Whitaker

**Affiliations:** 1 Department of Cardiology, St Thomas’ Hospital, London, United Kingdom (K.A., R.B., J.W.).; 2 Department of Congenital Heart Disease, Evelina London Children’s Hospital, United Kingdom (S.C., W.R., T.V., E.R., V.Z., J.M.S.).; 3 Department of Women and Children’s Health, King’s College London, United Kingdom (A.d.M.).; 4 British Heart Foundation Centre of Research Excellence, School of Cardiovascular and Metabolic Medicine and Sciences, London, United Kingdom (A.d.M.).; 5 School of Biomedical Engineering and Imaging Sciences, King’s College London, United Kingdom (V.Z., J.W.).; 6 Faculty of Life Sciences and Medicine, King’s College London, United Kingdom (R.B.).; 7 Department of Medical Genetics, Royal Brompton Hospital, London, United Kingdom (T.H.).; 8 Department of Medical Genetics, St George’s University of London, United Kingdom (T.H.).; 9 Institute of Reproductive and Developmental Biology, Imperial College London, United Kingdom (C.W.).

**Keywords:** atrioventricular block, bradycardia, death, sudden, cardiac, fetus, tachycardia

## Abstract

**BACKGROUND:**

Long QT syndrome (LQTS) is primarily an inherited condition associated with the risk of sudden cardiac death. Due to variable phenotypic expression, a prolonged QT interval on a 12-lead ECG is not always present. LQTS may present in the fetus with persistent bradycardia, including sinus bradycardia or functional 2:1 atrioventricular block. We report our experience of persistent fetal bradycardia prompting parental assessment for congenital LQTS.

**METHODS:**

From January 1, 2018 to November 1, 2023, 20 parents (20 mothers; 20 fathers) of fetuses presenting with persistent bradycardia and suspected congenital LQTS were assessed. Autoimmune-mediated atrioventricular block, diagnosed in the presence of maternal anti-Ro/anti-La antibodies, and fetuses with ventricular tachycardia were excluded. Parental ECGs were acquired in the remainder, with comprehensive evaluation, including genomic testing, performed in 12 mothers and 11 fathers.

**RESULTS:**

Among 20 fetuses, 16 had sinus bradycardia and 4 had 2:1 atrioventricular block (intermittent=2; persistent=2). Pathogenic LQTS genetic variants were found in 11 fetuses (*KCNQ1*=8; *KCNE1*=1; *KCNH2*=1; *CALM2* [calmodulin 2]=1), 9 mothers (*KCNQ1*=7; *KCNE1*=1; *KCNH2*=1) and 1 father (*KCNQ1*=1). Maternal corrected QT interval was higher in those with pathogenic variants compared with those who did not undergo genomic testing (456.9±11.6 versus 425.9±28.7 ms, *P*=0.009) but <400 ms in the paternal carrier. After review, 5 mothers with pathogenic variants were commenced on β-blockers (prepartum=4; postpartum=1). Provocation testing with a treadmill exercise test led to the initiation of β-blockade postnatally in one further case.

**CONCLUSIONS:**

The first indication of parental LQTS may be persistent fetal bradycardia. This should prompt consideration of this diagnosis even with a normal maternal corrected QT interval and lead to the initiation of specific management strategies for pregnancy, delivery, and the postpartum period before the results of genomic testing are available.

**WHAT IS KNOWN?**

- Long QT syndrome (LQTS) may present in the fetus as persistent bradycardia.
- The diagnosis of fetal LQTS is challenging, as fetal magnetocardiography, the reference standard to diagnose LQTS, is not routinely used and is only available in a small number of international centers.

**WHAT THE STUDY ADDS**

- Persistent fetal bradycardia may be the first and only indicator of parental LQTS.
- Of the 10 parents newly diagnosed with LQTS, none fulfilled the National Health Service England criteria for genomic testing, suggesting that the current reference standard clinical assessment tool is insensitive.
- We propose expansion of the criteria for genomic testing for LQTS pathogenic variants in adults to include isolated persistent fetal bradycardia and, where there is sufficient clinical suspicion of LQTS, consideration of β-blocker initiation and specific management strategies for pregnancy, delivery, and the high-risk postpartum period before the results of genomic testing are available.

Long QT syndrome (LQTS) is primarily an inherited disorder characterized by prolonged ventricular repolarization, which may manifest as a lengthened QT interval on a surface ECG. Affected individuals may be predisposed to the development of ventricular arrhythmias and are at increased risk of sudden cardiac death (SCD). It has a prevalence of 1 in 2000 and is associated with pathogenic variants identified in up to 17 genes.^1^ The most common pathogenic variants are loss-of-function mutations in *KCNQ1* (LQT1) and *KCNH2* (LQT2) or gain-of-function mutations in *SCN5A* (LQT3).^2^ Diagnosis of LQTS is based on clinical, electrocardiographic, and genetic factors, captured by a LQTS diagnostic criteria score, known as the Schwartz score.^1^ A consensus statement endorsed by the Heart Rhythm Society, European Heart Rhythm Association, and Asia-Pacific Heart Rhythm Society proposed that congenital LQTS is diagnosed when an individual has Schwartz score ≥3.5 or the corrected QT interval (QTc) is persistently >500 ms in the absence of QT-prolonging drugs or electrolyte abnormalities.^3^ Subsequent guidelines from the European Society of Cardiology recommend diagnosis with Schwartz score >3 or when QTc is persistently >480 ms in the absence of another cause.^4^ Both agree that LQTS should be diagnosed in the presence of an associated pathogenic variant. In addition to monogenic forms of congenital LQTS, there are individuals with less common and in some cases unidentified genetic causes, and a group with acquired LQTS, in whom a range of contributing genetic factors is likely relevant.^1^ Due to variable phenotypic expression, individuals with an LQTS-mediating pathogenic variant may have a normal QTc on a resting ECG. This is most common in LQT1, where 36% of patients do not demonstrate QTc prolongation on the surface ECG, compared with 19% of those with LQT2 and 10% of those with LQT3.^5^ Therefore, despite cardiac screening a diagnosis of LQTS may be missed and ancillary tests such as provocation testing which may unmask a concealed phenotype or genomic testing are frequently not undertaken. The QTc interval may increase during puberty, the postpartum period, after exercise, or after the initiation of certain medications.^1^ A LQTS diagnosis is, therefore, important to avoid QT-prolonging medications and to guide risk stratification and treatment, especially in pregnant mothers, where a diagnosis will have implications both for themselves and the fetus.

In utero diagnosis of fetal LQTS presents a diagnostic challenge. QT prolongation may be identified using fetal ECG or fetal magnetocardiography, the latter being the reference standard.^6^ Neither is routinely used in clinical practice, as these are only available in a small number of international centers. The left ventricular isovolumetric relaxation time and the left ventricular isovolumetric relaxation time indexed to the cardiac cycle on fetal echocardiography (normalized left ventricular isovolumetric relaxation time) have been proposed as alternative surrogate measures of the QT interval,^7^ but assessment is not possible if the presenting rhythm is 2:1 atrioventricular block and the sensitivity is suboptimal.^7–9^

The first indication of fetal LQTS may be persistent bradycardia. Given the potentially large pool of individuals with undiagnosed LQTS due to either lack of previous screening investigations or mild parental phenotype, the first indication of parental LQTS may, therefore, also be persistent fetal bradycardia. Identification of previously undiagnosed parental LQTS has important implications for safe maternal management during pregnancy and allows appropriate delivery plans to be made and implemented. We describe our experience of a cohort of parents who underwent evaluation for LQTS prompted by the identification of persistent fetal bradycardia. In each case, suspicion or confirmed diagnosis of maternal LQTS led to the development of specific management strategies for pregnancy, delivery, and the postpartum period.

## Methods

The authors declare that all supporting data are available within the article and its Supplemental Material. This study received institutional clinical governance approval (audit number 15423). Informed consent was not required. A retrospective review of electronic health records was performed to identify referrals for fetal bradycardia or referrals coded with a rhythm outcome or diagnosis of bradycardia or heart block at the Fetal Cardiology Unit (Evelina London Children’s Hospital, Guy’s and St Thomas’ National Health Service Foundation Trust) between January 2018 and August 2023.^8^ A free text search of scan reports and consultation comments for the term brady was performed to identify further cases. Fetuses with supraventricular tachycardia, ventricular tachycardia (VT), complete heart block, or ectopic beats were excluded, as well as those with major congenital heart disease, autoimmune-mediated atrioventricular block (secondary to maternal anti-Ro/anti-La antibodies), or a known cardiomyopathy.

Demographics, medical history, family history, and cardiac investigations were reviewed from the parental (mother and father of the affected fetus) electronic health record. Circumstances and outcome of delivery were obtained from a combined review of the parental and fetal electronic health record. Fetal heart rate and rhythm were assessed at serial time intervals using fetal echocardiography as per the institutional standard of care, with fetal bradycardia defined as 2 SD below the mean for gestational age.^6^ 12-lead ECGs were obtained from both parents of the affected fetus to assess QT interval, corrected using Bazett formula. A QTc >460 and >450 ms was considered prolonged in adult females and males, respectively.

**Figure 1.**
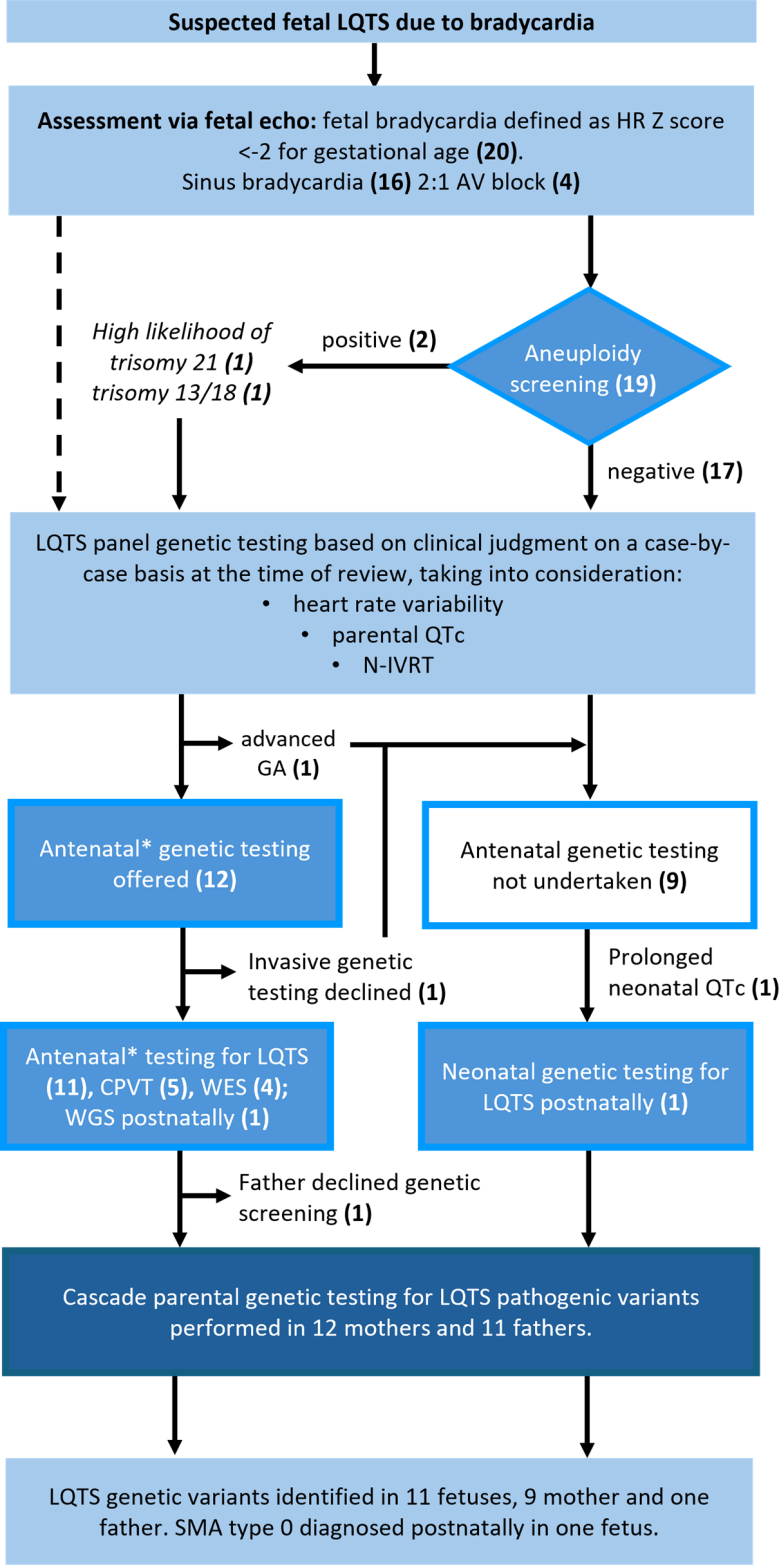
**Summary of our assessment of fetuses with persistent bradycardia.** AV indicates atrioventricular; CPVT, catecholaminergic polymorphic ventricular tachycardia; echo, echocardiogram; GA, gestational age; HR, heart rate; LQTS, long QT syndrome; N-IVRT, isovolumetric relaxation time as an index of the cardiac cycle; QTc, corrected QT interval; SMA, spinal muscular atrophy; WES, whole exome sequencing; and WGS, whole genome sequencing. *Antenatal genetic screening involved either amniocentesis or testing maternal blood.

Comprehensive parental evaluation was undertaken when there was clinical suspicion of LQTS from fetal findings. This comprised assessment by an adult cardiologist with expertise in the management of inherited arrhythmia syndromes and investigations which included 12-lead ECG, ambulatory ECG monitoring, and transthoracic echocardiography. Genomic testing for pathogenic LQTS variants using the Genomics England (https://www.genomicsengland.co.uk) recommended gene panel (labeled R127), which analyses the genes listed in Table 1,^10^ was offered at the discretion of the reviewing clinician. In cases where genomic testing was considered appropriate, counseling was undertaken. Counseling included information about the incidence of SCD attributable to channelopathies, the absence of a guideline-based indication in this circumstance as well as the issues covered by standard pretest genetic counseling. The pathogenicity of genetic variants was assessed using validated guidelines produced by the American College of Medical Genetics and the Association of Clinical Genomic Science.^11^ These guidelines are based on data from genomic databases, including the Human Gene Mutation Database, Single Nucleotide Polymorphism database, Genomic Aggregation Database, as well as the published literature, familial segregation studies, patch clamp data, and computational tools.^11^ Provocation testing using a treadmill exercise test was used to unmask the phenotype of patients with pathogenic LQTS genetic variants and a normal or borderline QTc interval and to guide initiation of β-blocker therapy in asymptomatic genotype-positive patients. Genomic testing was performed antenatally using amniocentesis, maternal and paternal blood samples, or postnatally using maternal, paternal, and neonatal blood samples (Figure 1). In isolated cases, the catecholaminergic polymorphic VT gene panel (labeled R129),^10^ whole exome sequencing, or whole genome sequencing was also performed to further investigate the underlying cause of fetal bradycardia (Table 1). The Schwartz score was used to determine the probability of a diagnosis of LQTS.^1,3,12^

**Table 1.**
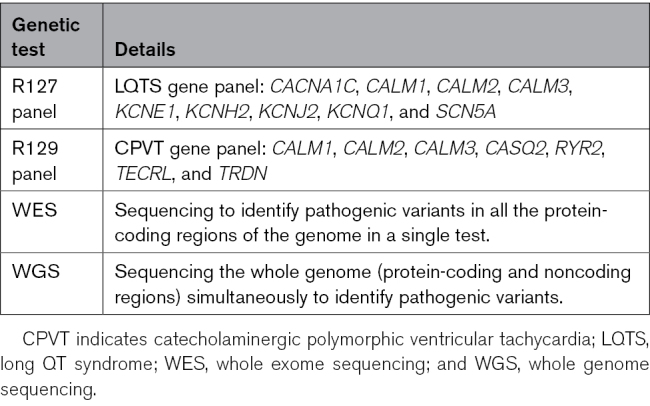
Summary of the Genomic Tests Used in Our Study Cohort

### Statistical Analysis

Statistical analysis was performed using Stata17 (StataCorp, 2021, StataCorp LLC; College Station, TX). Normally distributed data are presented as mean±SD and compared using a Student *t* test. Non-normally distributed data are presented as median (range or interquartile range) and compared using the nonparametric Mann-Whitney *U* test.

## Results

### Study Cohort

During the study period, 30 fetuses with bradycardia were identified. Two had autoimmune antibodies, whereas 8 had inherited structural abnormalities and were excluded. Fetal bradycardia was the primary reason prompting referral in 17 cases. Other reasons for referral included increased nuchal translucency (2 cases) and maternal congenital heart disease with a known ventricular septal defect (1 case). After assessment by a fetal cardiologist, 16 fetuses were found to have sinus bradycardia, and 4 fetuses had 2:1 block (intermittent in 2; sustained in 2; Figure 1). Parental demographics were recorded (Table 2). The mean parental age at the time of referral was 30.2 years. In all cases, there was no preexisting parental diagnosis of LQTS. Two additional fetuses presented with VT and were, therefore, excluded from our study. On retrospective review of referring hospital records, both fetuses had sinus bradycardia at their local hospitals, but it was not the presenting rhythm and did not prompt referral to our tertiary center.

**Table 2.**
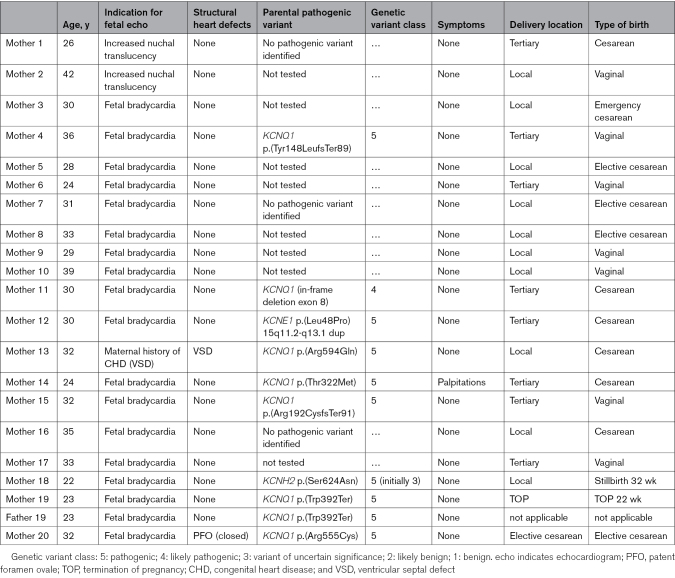
Demographic Characteristics of the Parents of Fetuses Referred to Our Tertiary Center With Persistent Fetal Bradycardia

In the following sections, maternal/paternal numbers and fetal numbers correspond to the family under consideration.

### LQTS Genotype-Positive Fetuses

A summary of the management of each fetus (including those with LQTS pathogenic variants, those with negative genomic testing, and those who were not tested) and their families is illustrated in Figures S1 and S2. LQTS genomic testing was undertaken in 12 fetuses (11 prenatally, 1 postnatally). Pathogenic variants were identified in 11 fetuses (*KCNQ1*=8, *KCNE1*=1, *KCNH2*=1, and *CALM2 [calmodulin 2]* =1) including 2 de novo mutations (*KCNQ1*=1 and *CALM2*=1). Fetuses carrying LQTS pathogenic variants with 1:1 atrioventricular conduction were noted to have a lower median fetal heart rate *Z* score than those who had negative genomic testing or who were not tested (−3.20 versus −2.35; *P*=0.03; Figure S3).

After delivery, all neonates with LQTS pathogenic variants except for fetus 12 had a prolonged QTc and were initiated on a β-blocker. None exhibited evidence of atrioventricular block. Fetus 16 underwent insertion of an implantable cardiac defibrillator due to a significantly prolonged postnatal QTc of 680 ms and subsequent identification of a pathogenic *CALM2* mutation known to be associated with a severe phenotype with a high risk of cardiac arrhythmias in early childhood.

Fetus 18, who had a *KCNH2* variant of unknown significance (maternally inherited) identified prenatally, was stillborn at 32 weeks. This variant was subsequently reclassified as pathogenic postmortem. The combination of an increased nuchal translucency, increased nuchal fold, and recurrent pleural effusions observed in fetus 18, however, is not typical of LQTS. There was also only sinus bradycardia observed on successive reviews with no evidence of fetal tachycardia or paroxysmal VT to explain the development of fetal hydrops and subsequent fetal demise. Whole genome sequencing identified a postmortem de novo *PACS1* pathogenic variant in fetus 18 in addition to the *KCNH2* variant identified prenatally. This variant is associated with Schuurs-Hoeijmakers syndrome, a condition characterized by neurodevelopmental delay, facial, cardiac, and gastrointestinal congenital anomalies, intellectual disability, and the development of pleural effusions.^13^

Two fetuses with Jervell and Lange-Nielsen syndrome were identified, both of whom were homozygous for a *KCNQ1* variant (fetuses 11 and 19). The former had a maternally inherited genetic variant, but the origin of the second allele remains unknown as the father declined genomic testing. The latter inherited 1 variant from each parent. After prenatal diagnosis, the mother of fetus 19 underwent termination of pregnancy at 22 weeks. All parents received genomic counseling regarding the likelihood of LQTS affecting their future pregnancies.

### LQTS Genotype-Negative Fetuses/Not Tested

Fetus 1 tested negative for LQTS and catecholaminergic polymorphic VT pathogenic variants on whole exome sequencing despite persistent 2:1 block on serial fetal echocardiograms from 23 to 32 weeks (fetal ventricular rate 65–77 bpm). Postnatally, the infant was diagnosed with spinal muscular atrophy type 0 (Figure 1). In the remaining 8 fetuses, genomic testing was not pursued, and therefore, the differential diagnosis may include other causes. Mothers 2 and 5 were offered, but declined genomic testing despite careful counseling on the risk of sudden death associated with LQTS. Mother 6 presented later in pregnancy such that prenatal invasive genomic testing was not offered. In the remainder (n=5), a clinical decision was made by the attending fetal cardiologist on a case-by-case basis not to pursue prenatal genomic testing for fetuses 3, 8, 9, 10, and 17. Factors taken into consideration included a normal parental QTc (fetuses 3, 8, 9, 10, and 17), normal fetal left ventricular isovolumetric relaxation time indexed to the cardiac cycle (fetuses 8, 9, 10, and 17), and a normal neonatal QTc (fetuses 3, 8, 9, 10, and 17).

All 8 fetuses also had a normal postnatal QTc, and without a clear phenotype, in the neonate genomic testing was deferred. Our previous practice was to follow-up these fetuses with serial ECGs and when old enough to perform an exercise ECG to review lying, standing, and recovery QT intervals. However, based on the high yield obtained from genomic testing for LQTS pathogenic variants in fetuses with isolated persistent bradycardia, our practice has evolved, and we have started to offer postnatal genomic testing to these families based on this phenotype alone.

### Parental Genetic Testing and Clinical Phenotyping

**Figure 2.**
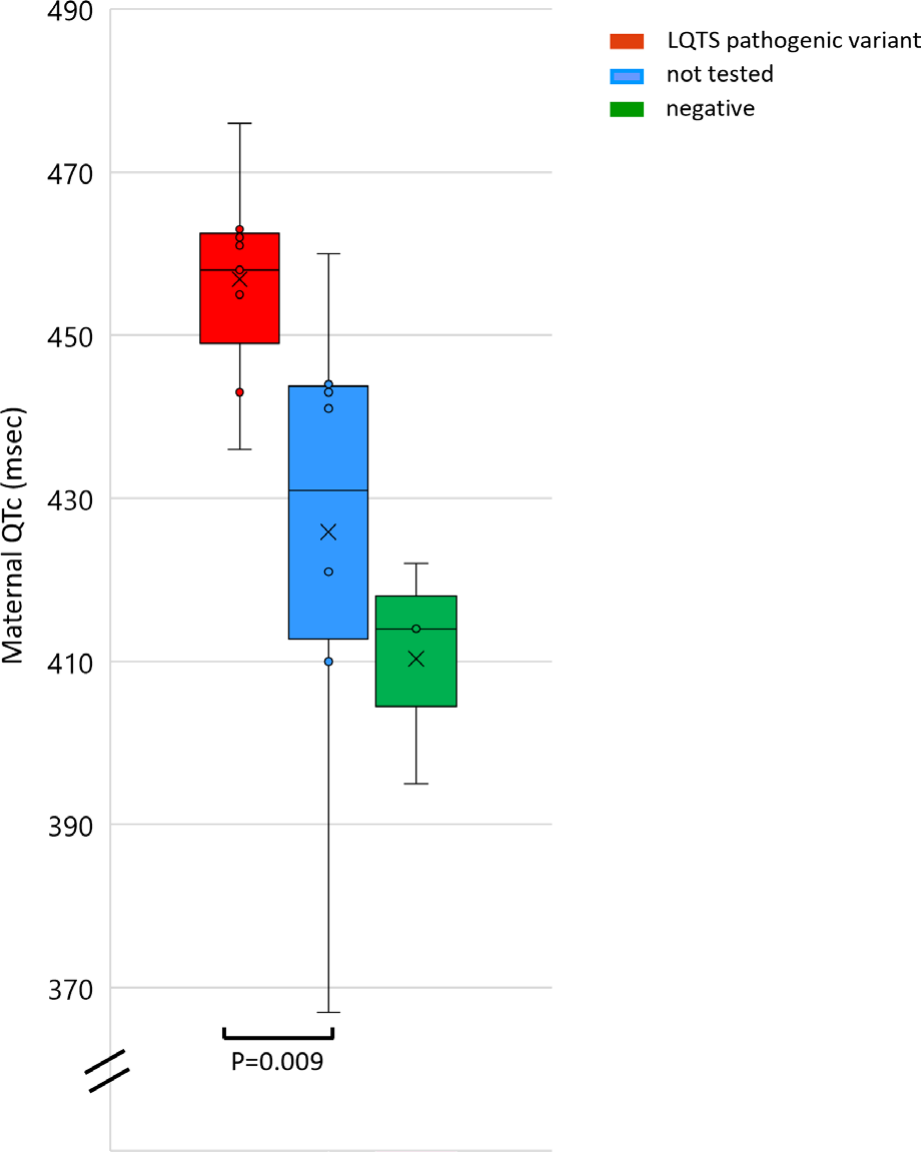
**Maternal corrected QT interval (QTc) interval in those with long QT syndrome (LQTS) pathogenic variants vs those not tested (456.9±11.6 vs 425.9±28.7 ms, *P*=0.009).** The mean QTc interval of mothers who tested negative for LQTS pathogenic variants was 410.3±13.9.

**Figure 3.**
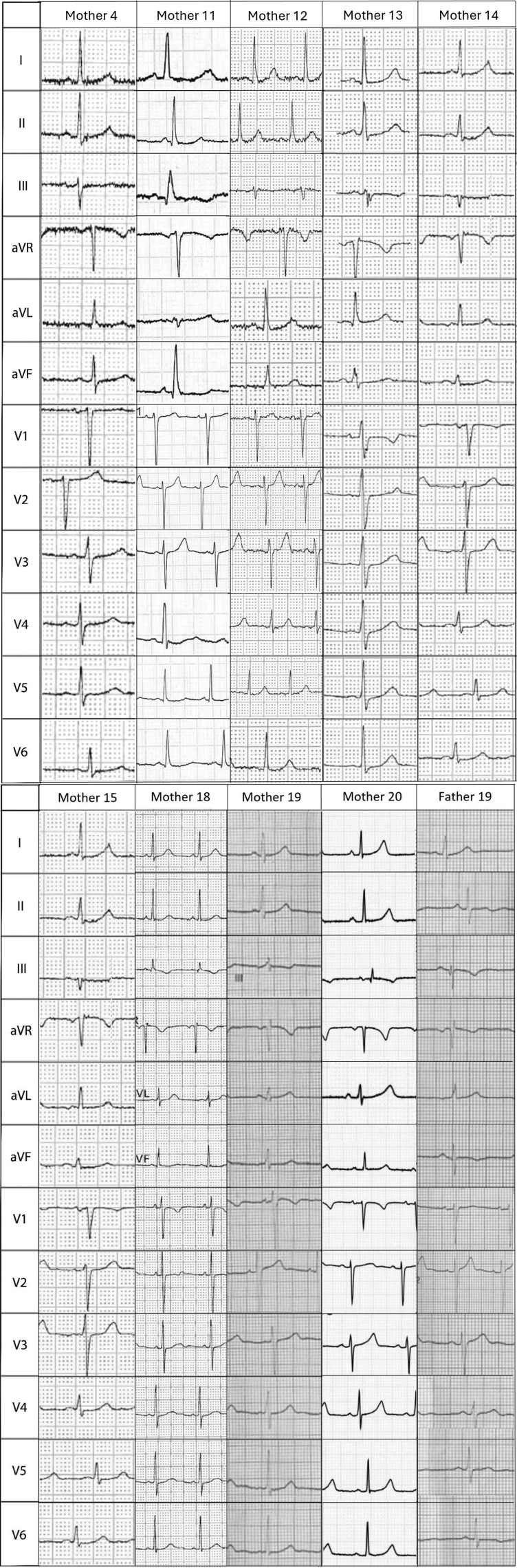
Electrocardiograms from parents carrying long QT syndrome (LQTS) pathogenic variants.

Genomic testing for LQTS was offered to 13 mothers and 13 fathers, and undertaken in 12 and 11, respectively. Pathogenic variants were identified in 9 mothers (*KCNQ1*=7, *KCNE1*=1, *KCNH2*=1) and 1 father (*KCNQ1*=1). The majority were asymptomatic except for 1 mother with a *KCNQ1* mutation who reported stress-induced palpitations prepartum and a syncopal episode postpartum. None had a family history of SCD. Four mothers with pathogenic LQTS variants had prolonged QTc on their resting ECG (460–470 ms: 3 [mothers 4, 11, and 14], ≥470 ms: 1 [mother 13]), whereas 5 had a QTc <460 ms (mothers 12, 15, 18, 19, and 20). Mean maternal QTc was significantly higher in those with a pathogenic variant compared with those who did not undergo genomic testing (456.9±11.6 versus 425.9±28.7 ms, *P*=0.009), but the paternal carrier had a QTc <400 ms (Figure 2). Mothers and fathers who tested negative for LQTS pathogenic variants had a mean QTc of 410.3±13.9 and 407.9±26.9, respectively. Bifid T-waves were present on the ECG in mother 18, and low-amplitude, peaked T-waves with a flat isoelectric segment were present in mother 13 (Figure 3A and 3B). Of those with pathogenic variants, the mean Schwartz score was 0.571 (Table 3). Overall, in this cohort, 5 mothers with a QTc <460 ms and without any other phenotypic features suggestive of LQTS were diagnosed with congenital LQTS after genetic testing, which was prompted solely by persistent fetal bradycardia.

**Table 3.**
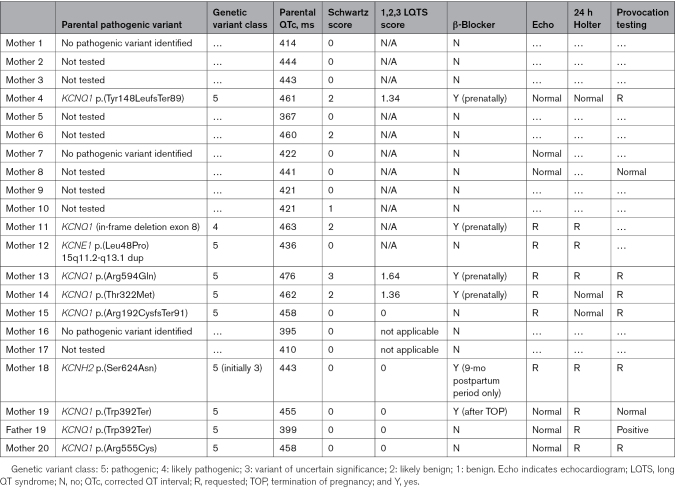
Further Investigations Performed in Parents Suspected of Having a LQTS Pathogenic Variant Prompted by the Identification of Persistent Fetal Bradycardia

### Management

All parents with a potential diagnosis of LQTS were counseled regarding the early use of oral rehydration therapy to replace fluid losses during episodes of diarrhea or vomiting and to attend the emergency department immediately after any episode of syncope. Mothers were advised to avoid QT-prolonging medications, including breastfeeding mothers who had tested negative for LQTS pathogenic variants, where there was suspicion of a de novo mutation in the fetus. This guidance is consistent with lifestyle advice routinely given to patients with LQTS. Specific recommendations regarding exercise also include the avoidance of swimming alone for individuals with a high clinical suspicion for LQT1.

β-Blockers (most commonly bisoprolol) were initiated during pregnancy in 4 mothers who had a confirmed genetic diagnosis of LQTS with a prolonged QTc >460 ms. A further 2 women were commenced on β-blockers postnatally. Mother 19, a *KCNQ1* carrier with a borderline QTc of 455 ms was commenced on β-blockade after a positive treadmill test. β-Blockers were used during the 9-month high-risk postpartum period in mother 18. There were no significant differences in the mean birth weight (2.95 versus 2.87 kg; *P*=0.06) between fetuses who had been exposed to β-blockers in utero compared with those who had not, and no cases of fetal hypoglycemia. After an episode of syncope, which occurred 2 years and 4 months after delivery, mother 14 was managed with nadolol.

### Delivery

In those with a genetically confirmed fetal or maternal LQTS diagnosis or a clinical suspicion for this diagnosis, prophylactic measures were implemented during the pregnancy to minimize the risk of maternal arrhythmia. These included the recommendation to avoid QT-prolonging medications, for example, oxytocin during induction,^14^ ondansetron,^15^ or anesthetic agents, such as sevoflurane.^16^ In addition, a bespoke management plan for delivery was implemented with involvement of adult inherited cardiac conditions and obstetric teams. Careful consideration was given to the appropriate location for delivery, which was ultimately determined by the estimated risk of fetal compromise, parental preference, and local center capacity. Ten mothers delivered at a local general hospital, whereas 8 were delivered at our institution, which provides a tertiary service for obstetric, pediatric, and adult cardiology specialties. Common to all birth plans was the use of cardiac telemetry during induction, proactive measurement and correction of electrolytes, and avoidance of QT-prolonging drugs. Eleven deliveries were by cesarean section. Although maternal LQTS did not change the mode of delivery, persistent fetal bradycardia was an important intrapartum consideration. A case-by-case approach was used by the obstetric team when planning the mode of delivery, taking into consideration both the fetal cardiac status and the safety of intrapartum monitoring. No arrhythmias occurred during delivery among the mothers of the 20 fetuses with suspected or genetically confirmed LQTS.

### Follow-Up Investigations

Consistent with the usual standard of care, 24-hour ambulatory ECG and echocardiography were offered to all parents with a suspected new diagnosis of LQTS arising from the pregnancy (Table 3). Overall, 30% (3/10) and 50% (6/12) of individuals, respectively, completed these investigations, the results of which were unremarkable (Figure S4). Nine parents were offered provocation exercise treadmill testing according to the Bruce protocol,^17^ which was undertaken in 3. QT prolongation was observed with a QTc of 587 ms 4 minutes into recovery in mother 19, whereas 2 parents had a normal response to exertion. Wider familial screening led to identification of LQTS pathogenic variants in 3 of 7 siblings tested.

## Discussion

We present a single-center experience of persistent fetal bradycardia prompting parental diagnosis of previously unidentified congenital LQTS. The main findings from this study are that (1) persistent fetal bradycardia may be the only indication of previously undiagnosed parental LQTS; (2) the current reference standard clinical assessment tool used for the diagnosis of LQTS was insensitive in the group of patients in whom a genetic diagnosis of LQTS was made; and (3) after exclusion of an autoimmune or a significant coexisting fetal syndrome or structural abnormality, selective genomic testing produced a high yield for parental pathogenic variants associated with LQTS.

Among 20 fetuses who presented with persistent bradycardia, mostly sinus bradycardia, 12 mothers and 11 fathers underwent genomic testing. Ten of these parents, including 9 mothers, were diagnosed with genetically mediated LQTS. With the exception of 1 mother with stress-induced palpitations before conception, all parents with LQTS genetic variants were asymptomatic, none had a family history of LQTS, and 2 mothers (mother 12 and 18) and 1 father (father 19) with LQTS genetic variants had a normal QTc on their resting ECG. Only 1 patient had a Schwartz score ≥3.

The first presentation of congenital LQTS in the adult population may be unheralded ventricular arrhythmia which, although infrequent, is potentially catastrophic. The risk of SCD in LQTS can be mitigated by implementation of simple lifestyle measures, including avoidance of arrhythmic triggers and QT-prolonging drugs.^18^ Crucially, for patients with frank QTc prolongation, medical therapy with a β-blocker may be initiated with minimal risk during pregnancy. These strategies can be commenced safely before genomic testing in patients in whom a LQTS diagnosis is being considered.

As per recommendations from the European Society of Cardiology, we used a shared decision-making approach regarding participation in high-intensity sport with appropriate risk stratification, taking into consideration the type and nature of exercise, the type of pathogenic variant, the QTc interval, symptoms, and whether there was previous arrhythmia or cardiac arrest.^19^ The American Heart Association and the American College of Cardiology in contrast are less restrictive and allow participation in competitive sports to be considered (with the exception of competitive swimming in LQT1) provided precautionary measures are undertaken including avoiding QT-prolonging medication, electrolyte replacement, adequate hydration and the inclusion of an automated external defibrillator as part of the athlete’s personal sports gear.^20^

### Genomic Testing

Currently, fetal bradycardia is not identified as an indication for LQTS genomic testing in the fetus or neonate.^10^ Among the parents in this cohort, only 1 adult had a recognized indication for genomic testing, and none fulfilled the National Health Service England criteria for genomic testing for LQTS^10^ (Figure S5).

These data prompt consideration of the expansion of the indications for LQTS genomic testing to include isolated persistent fetal bradycardia. The understanding of LQTS genomics is evolving, and these data raise the possibility of a group of undiagnosed LQTS patients with subtle or absent phenotypes, in whom the optimal risk stratification and management strategies are unknown. Identification and characterization of this group would contribute to the development of our understanding of the relevance of these genetic findings across wider populations. For individual patients, finding a pathogenic genetic variant may inform gene and variant-specific risk stratification to guide long-term management and facilitate shared decision-making around the management of risk and uncertainty. For some, it may prompt targeted therapy such as the use of mexiletine in patients with LQT3^21^ and genotype-specific advice regarding triggers, such as avoidance of strenuous swimming or diving into cold water in patients with LQT1.^22^

Jervell and Lange-Nielsen syndrome, a highly malignant form of LQTS, was identified in 2 cases.^23^ Preterm identification at 20 weeks in 1 case permitted an informed discussion and resulted in a parental decision to pursue a termination of pregnancy after genomic counseling and advice regarding reproductive options for future pregnancies.

One patient in this series (mother 18) was initially found to have a genetic variant of uncertain significance in the *KCNH2* gene. Detection of such a variant presents a clinical challenge. The mother was asymptomatic with a normal QTc, and after careful discussion, the patient and care team reached an agreement to discontinue β-blockers beyond the postpartum period. After familial segregation studies and a multidisciplinary review of patch clamp data, this variant was subsequently reclassified as pathogenic, and β-blocker therapy was restarted. Any proposal to expand genomic testing criteria is likely to increase the incidence of this type of clinical dilemma, which reinforces the importance of thorough counseling before genomic testing, and the possibility of identifying such a variant should be considered by the patient before consent is given for the test. In the context of these data, which indicate likely greater prevalence of genotype-positive/phenotype-negative individuals than previously appreciated, particular emphasis may also be given to the uncertainty of the implications of identifying even a pathogenic variant in the absence of clinical phenotype. A shared decision-making approach when deciding whether to undergo a genomic test is, therefore, of the greatest importance.

### Risk of Sudden Death During Pregnancy

A lower incidence of cardiac events has been reported in women with LQTS during pregnancy compared with preconception.^24^ The risks, however, may increase in patients who experience a complication of pregnancy, such as hyperemesis, which can result in severely restricted oral intake and significant electrolyte derangement leading to an elevated risk of ventricular arrythmia.^25^

The 9-month postpartum period in women with LQTS is particularly high risk, with a 2.7 times greater risk of a cardiac event compared with preconception, with women with LQT2 at greatest risk.^25^ Suggested contributory factors include a rapid decline in cardiac output in the early postpartum period due to reduced myocardial contractility and preload.^26^ Rapid postpartum decline in estrogen levels, altered sleep patterns, the stress associated with caring for a newborn infant, and accompanying loud auditory stimulus may also predispose patients with LQT1 and LQT2 to malignant arrhythmia.^27^

These data indicate that the risks of SCD in LQTS may increase during and soon after delivery. It, therefore, seems reasonable, in some patients, to recommend simple low-risk lifestyle interventions before the completion of maternal genotyping and phenotyping, and this has become our standard practice. It is acknowledged that the event rate is low, perhaps due to mild or concealed phenotypes in this particular group, and we emphasize that our data contain no indication that the introduction of these measures has affected outcomes.

### Use of β-Blockers

β-blockers remain the first-line pharmacological therapy in patients with LQTS pathogenic variants.^28^ All 4 patients with a prolonged QTc >460 ms were commenced on β-blockers. β-Blockers were used in the high-risk postpartum period in a patient with LQT2 and another patient with positive provocation testing. Heart Rhythm Society and European Society of Cardiology guidelines recommend the use of β-blockers as a class I indication in individuals with LQTS who have symptoms or who are asymptomatic with QTc prolongation >470 ms.^29^ QTc prolongation is associated with a substantially higher risk of SCD among LQTS pathogenic variant carriers; however, even in the absence of QTc prolongation, genotype-positive (phenotype-negative) individuals are at a higher risk than family members without a genetic variant.^29^ As such, consideration of a β-blocker is recommended in all pregnant women with LQTS pathogenic variants.^29^

Bisoprolol was the β-blocker primarily used in our cohort due to the mild phenotype in the majority of patients as well as its known cardioselectivity and safety profile in pregnancy. One patient (mother 14) was subsequently switched to nadolol after an episode of stress-induced syncope over 2 years after delivery. Although there are no randomized controlled trials comparing the relative efficacy of various β-blockers in LQTS, there is a consensus among experts based on retrospective analysis of available patient cohorts that nadolol is the preferred first-line agent. Abu-Zeitone et al^30^ demonstrated that although atenolol, metoprolol, propranolol, and nadolol all had similar efficacy at reducing the risk of a first cardiac event in patients with LQT1, only nadolol was effective in patients with LQT2. More recently, Mazzanti et al^31^ showed in a large registry of patients with LQTS that nadolol was the only β-blocker associated with a reduction in the 5-year risk of a life-threatening cardiac event, irrespective of genotype. β-blocker use in pregnancy requires caution, given the possibility that fetuses exposed in utero to β-blockers are at risk of intrauterine growth restriction, neonatal hypoglycemia, and bradycardia.^32–34^ There was no evidence of these effects in our cohort.

### Maternal Inheritance

There was a high incidence of maternal transmission of LQTS pathogenic variants to the fetus, but the cohort size presented is too small for any conclusions to be drawn from this observation. We note that a pedigree study has suggested that the inheritance of LQTS pathogenic variants does not follow a classic Mendelian pattern but rather shows preferential transmission of pathogenic variants from mothers to daughters, leading to an unbalanced female prevalence of LQTS.^35^ There may additionally be positive selection of LQTS pathogenic variants during gametogenesis or fertilization, which skews transmission, leading to a greater incidence of pathogenic variants among descendants of affected families.^35^

### Genomic Testing

Although genomic testing can be a powerful tool to confirm a diagnosis of LQTS, the prevalence of variants of uncertain significance means its judicious use is essential to avoid an erroneous diagnosis, the generation of anxiety and uncertainty, as well as inappropriate treatment. There is currently no formal consensus on fetal genomic testing in the context of isolated persistent fetal bradycardia, and as such, early in our experience, the justification for offering genetic testing to these fetuses was less clear. Based on our increasing appreciation of the association between persistent fetal bradycardia and otherwise silent LQTS, our practice has evolved such that genomic testing is now considered and offered to those presenting with isolated fetal bradycardia. Furthermore, postnatal genomic testing is being offered to fetuses with persistent bradycardia who were not tested antenatally, as well as exercise provocation testing once of a suitable age. This approach averts potentially missed diagnoses with immediate prognostic implications and allows prompt initiation of a β-blocker in genotype-positive infants and parents should a phenotype become evident. It will also help us understand the specificity of persistent fetal bradycardia as an indicator of fetal LQTS and inform our future practice to ensure the sensible use of genetic testing.

### Alternative Causes of Fetal Bradycardia

Persistent fetal bradycardia may be the first presentation of other inherited conditions. Fetus 1 had fixed 2:1 atrioventricular block with a negative LQTS gene panel and was antenatally diagnosed with spinal muscular atrophy type 0 after whole genome sequencing testing. Spinal muscular atrophy type 0 may be associated with bradycardias and autonomic effects, which is the plausible cause in this case.^36^ Equally, bradycardia may reflect overall fetal wellbeing, such as was the case in fetus 2, who was postnatally diagnosed with trisomy 21. After delivery, they were also found to have an atrial septal defect.

We acknowledge that other inherited cardiac channelopathies, such as *RYR2* [ryanodine 2] and *HCN4* [hyperpolarization activated cyclic nucleotide gated potassium channel 4] pathogenic variants, may present with fetal bradycardia.^9^ The former is implicated in catecholaminergic polymorphic VT, whereas the latter conducts the hyperpolarization-activated current in the sinoatrial node.^9^ Future work could involve the utilization of broader genomic analysis to identify other potential inherited conditions in fetuses who test negative for LQTS with no alternative cause identified for persistent bradycardia.

### Limitations

This is an observational study and includes a small cohort of patients. Referral bias may have contributed to the large proportion of LQTS variants in fetuses presenting with bradycardia. Several genotype-positive parents were lost to follow-up after delivery and did not complete ancillary investigations for comprehensive phenotypic evaluation. Possible reasons for this include low prioritization of appointments when asymptomatic, travel costs, and difficulty arranging childcare with a newborn. These practical difficulties have important implications for a patient group who are often primary caregivers for infants, who may have to travel long distances to visit a tertiary center, and in whom assessment is taking place after a significant life event.

Establishing a patient pathway that attempts to overcome the unique difficulties young parents, in particular mothers, face around this time while ensuring equitable access to diagnostic investigations with potential prognostic significance is recommended, given the results presented in this study. Our observations suggest a pragmatic approach to risk management during diagnostic evaluation, acknowledging that clinical decision-making may have to take place before completion of all intended investigations. A pathway that facilitates flexible and appropriate timing for assessment, counseling, investigations, and interventions around the competing priorities these patients face represents an institutional priority.

### Conclusions

Fetal bradycardia may be the first and only indication of parental LQTS. In this cohort, cascade screening prompted by recognition of persistent fetal bradycardias led to a high incidence of new parental diagnoses of LQTS. Thorough parental phenotyping and LQTS genomic testing should be undertaken in the presence of isolated fetal bradycardia, even in the absence of QTc prolongation. Prescription of simple lifestyle measures, specific precautions during delivery, and, in a subset of mothers, prophylactic β-blockade to reduce the risk of a cardiac event may be justified in some patients while waiting for results of clinical investigations and genomic testing. Our understanding of the genetics of LQTS continues to evolve, and optimal management of asymptomatic genotype-positive patients with mild/negative phenotypes remains unclear.

## ARTICLE INFORMATION

### Acknowledgments

We acknowledge the support of the Wellcome Engineering and Physical Sciences Research Council (EPSRC) Centre for Medical Engineering at King’s College London (WT 203148/Z/16/Z).

### Sources of Funding

S. Chivers is funded by Diabetes UK, Sir George Alberti Fellowship, grant reference: 21/0006277. Dr Rosenthal receives travel support from BVM Medical.

### Disclosures

Dr Bastiaenen received consulting fees from Bristol Myers Squibb and Pfizer. The other authors report no conflicts.

### Supplemental Material

Figures S1–S5
